# A Case of Mural Pregnancy

**Published:** 1885-01

**Authors:** William H. Byford

**Affiliations:** Professor of Gynecology, Rush Medical College, Chicago


### THE CHICAGO
### MEDICAL JOURNAL AND EXAMINER.

VOL. L. JANUARY, 1885. NO. I.

### ORIGINAL COMMUNICATIONS.

A CASE OF MURAL PREGNANCY. *By* WILLIAM H. BYFORD, *M. D., Professor of Gynecology, Rush Medical College, Chicago.*

[*Read before the Chicago Gynecological Society, Dec. 19th,* 1884.]

The history of this case is so obscure on account of the ignorance of the patient as to make it almost valueless. In substance she gave it about as follows:

She was twenty-eight years of age, had been married seven years, and had one child, now six years old. She supposed she became pregnant again in February, 1883. In April she was very much fatigued and had hemorrhage, which continued until May 9th—about four weeks. After that she had no hemorrhage until October 14th.

At that time there occurred a discharge of yellow fluid, which she estimated at about one gallon in quantity. This was followed by a putrescent sero-sanguineous discharge for about three months. In January, 1884, a large brownish mass with very fœtid odor came away. Soon after this, the menses were re-established and continued until July. In May, she was quite large, and had bearing down pains. She had no pain after July.

She entered the hospital October 6th, 1884. October 18th, she was tapped, and about four quarts of thick, tenacious fluid, strongly resembling such as we often draw from an ovarian tumor. It coagulated on the addition of nitric acid and on boiling.

I made a microscopic examination of this fluid, assisted by Dr. Tilley, but could not derive any definite information from it. It did not contain the Drysdale cell.

Laparotomy was performed, the fœtus and placenta removed without any difficulty or considerable hemorrhage.

In order to make a cavity that might be drained well, it was considered best to remove the uterus, as the specimen will show.

The operation was done October 13th, and the patient did not rally, but died in about twenty-four hours.

The patient before the operation was greatly reduced in strength on account of her protracted suffering.

If I had been as well informed on the subject of this kind of pregnancy, as I was after this observation, I would have operated through the vagina, instead of performing laparotomy.

This is the second case of mural pregnancy, I have seen within about five years. The first case was reported to this society, a résumé of which is as follows:

The patient was in labor and was moribund when I first saw her. She had been in labor until exhausted. There was no difficulty in making a diagnosis. The head was low down in the pelvis, almost on the perineum. The os uteri was well-nigh inaccessable behind and above the symphysis pubis. The body of the uterus could be felt, somewhat larger than natural, in the lower and anterior part of the abdomen, attached to the tnmor containing the fœtus. The fœtus could be felt through the abdominal walls, surrounded by a thick involucrum, apparently as thick as the uterine walls.

The limbs and other portions of it were distinguishable.

When dissected, the sac in which the fœtus and membranes were contained, consisting of the amnion and chorion with the liquor amnii, was found to be formed of a thick layer of large muscular fibers. These latter were continuous with the fibers of the posterior wall of the uterus, and extended upward, downward and laterally, invading the whole of the wall of the sac in every direction. These fibers resembled in every way those found in the pregnant uterus at full term. The enveloping fibrous structure was much thinner than the walls of the uterus, and the fœtus could be indistinctly seen as it lay enclosed in it and its proper membranes. The Fallopian tubes and ovaries lay beside the lower portion of the sac. The posterior wall of the uterus formed a part of the anterior wall of the containing cavity. As viewed before and during the section, it gave one the idea that the sac, containing the fœtus, had been developed by the growth of a detached layer of the muscular fibers of the posterior wall of the uterus; and that is what I think was the fact, and might be explained in the following manner: the fructified ovum had made its way down the tube to the intramural portion, when it was arrested, but continued to grow, and, as it grew, it produced upon the uterine structures the same trophic influences, that it would have done, had it been contained in the natural cavity. When this process was begun, it was continued by the wonderful energies of development, which takes place in the uterus and could occur nowhere else in the body. It was, in fact, an ectopic, *uterine* pregnancy. I do not believe there was any structural violence done at the seat of development in the sense of tearing away the layer of fibers in which it grew. But the ovum, having insinuated itself beneath the outer layer of muscular fibers, they grew sufficiently to make the large cavity containing the ovum. The fœtus was thus developed within the uterus, although not in the uterine cavity, and, because of this, the pregnancy more nearly resembled normal gestation than any other form of ectopic pregnancy.

The resemblance is especial in the position and presentation of the fœtus, at term deep in the pelvic cavity behind the vagina. The head in this case was fixed by the concentric contraction of the uterine fibers, by which it was surrounded, and could be easily outlined as it lay there crowded behind the posterior vaginal wall.

The specimen here presented is much less perfect than the one described, because of the numerous effects wrought upon it by the great length of time in which retrograde changes had been going on and the mutilations produced by our efforts at its removal. It is, however, sufficiently so to enable you to trace nearly all the conditions presented by the other.

The changes alluded to greatly obscured the nature of the case and made our diagnosis doubtful. The walls were thickened and indurated; the fluid contained in the sac was viscid and contained much albuminoid material, rendering it impossible to distinguish the shape or even presence of the fœtus. We believed it to be an extrauterine pregnancy, but could not demonstrate the fact without incision, and it was not until the whole was removed that we could determine the character of the pregnancy.

The treatment of these cases ought to be considered apart from that of extrauterine pregnancy, especially at term. It is always a matter of special consideration, in connection with each case as it presents itself, whether the removal of the fœtus at term in extrauterine gestation should be attempted; whether laparotomy for the delivery does not subject the patient to greater hazard than to leave her to the reparative process immediately instituted for her preservation.

The dangers of laparotomy are greatly increased by our inability to remove the placenta. The surface to which it is attached has no contractile power, so that the divided vessels are left patent. If hemorrhage does not immediately prove fatal, the blood is a source of sepsis that must almost certainly destroy the patient.

In passing, I might say that I believe laparotomy would more likely be successful, if performed some days after the death of the child. My reason for advising the postponement of the operation is that when the circulation in the placenta ceases, the blood-vessels collapse, do not contain blood, and the danger in separating the placenta, for its removal, would be reduced to a minimum.

In the cases of ectopic or interstitial uterine pregnancy, the fœtus may, I think, be easily removed through the vagina. An incision made through the posterior vaginal wall would completely uncover the presenting part and enable us to apply the forceps, or attack it with the perforator and crotchet, as in ordinary labor. After the removal of the fœtus, the placenta should be allowed to separate spontaneously. It might then be taken from the cavity without any special danger.

I arrive at this latter conclusion from the reflection that that organ is attached to a fibrous base, capable of contraction. It is, in fact, a base of uterine muscle. Another fact, of great value in this connection, is that the cavity thus opened does not communicate with the peritoneum, and consequently may be thoroughly drained and disinfected by washes through the vaginal opening made for the delivery of the fœtus.

Although I have not had a suitable opportunity to put my ideas upon the subject in practice, I would unhesitatingly do so as affording the most plausible method of giving the patient a chance for her life.

Since writing the above, I have seen a case very imperfectly reported in the "*Annales de Gynecologie*" for July, 1884. It occurred in the practice of Mr. Matheson, of England, which seems to me to have been the execution the plan of delivery here indicated.

Mr. Matheson reported the case to the London Obstetrical Society.

The article is headed: "Extrauterine Pregnancy. The Extraction of a Living Fœtus through the Vagina."

The head was in the pelvis, covered by the greatly distended posterior wall of the vagina. An incision was made upon it, and the face was made to present. The forceps was applied, and the extraction was effected without difficulty.

The child was in a state of suspended animation, but upon the application of restorative measures, was resuscitated. It weighed nine pounds. The placenta came away without dangerous hemorrhage, and, while it did not seem necessary, a sponge wet with the perchloride of iron was introduced into the cavity.

The patient recovered without any alarming symptoms.

It would not seem that the author suspected his case to be one of interstitial pregnancy. During the discussion, that followed, only one of those, who participated in it, expressed the opinion that it was one of that kind.

Mr. Griffith thought it was either interstitial pregnancy, or one, in which the fœtus was developed in one portion of a double uterus.

I have made no attempt to look up the literature of this form of ectopic pregnancy. My object has been to give as concise a description of its anatomy as was consistent with accuracy, and submit my reflections upon the nature and treatment of it.

[The cut, illustrating Professor Byford's first case, will appear in our next issue, in connection with the report of the Pathologist, Professor Christian Fenger, M. D.—ED.]

